# Clinical Landscape and Perception of Surgical Neuro Oncology among Neurosurgical Resident Trainees of Pakistan: A Nationwide Analysis

**DOI:** 10.12669/pjms.41.13(PINS-NNOS).13446

**Published:** 2025-12

**Authors:** Syed Haider Hassan, Haseeb Mehmood Qadri, Talha Sajid, Raana Shahid, Ismaeel Khalid Iqbal, Zermina Tanvir, Amal Mahmood

**Affiliations:** 1 Syed Haider Hassan, MBBS, Punjab Institute of Neurosciences, Lahore, Pakistan; 2 Haseeb Mehmood Qadri, MBBS, Punjab Institute of Neurosciences, Lahore, Pakistan; 3 Talha Sajid, MBBS, Punjab Institute of Neurosciences, Lahore, Pakistan; 4 Raana Shahid, MBBS, Punjab Institute of Neurosciences, Lahore, Pakistan; 5 Ismaeel Khalid Iqbal, MBBS, Punjab Institute of Neurosciences, Lahore, Pakistan; 6 Zermina Tanvir, MBBS, Nishtar Hospital Multan, Pakistan; 7 Amal Mahmood, Medical Student, Aga Khan University, Karachi, Pakistan

**Keywords:** Residency, Hospital Oncology Service, Neurosurgeries, Training Programs, Pakistan

## Abstract

**Objective:**

To assess the neurosurgical residents’ perspectives on surgical neuro-oncology specialty training in Pakistan.

**Methodology:**

A nationwide survey-based observational study was conducted among neurosurgery residents registered with the accredited residency programs in Pakistan. A structured questionnaire assessing knowledge, training, mentorship, and institutional support in surgical neuro-oncology was disseminated via Google Forms (Google Inc., USA). Data collection took place from January to March, 2025 across both public and private sector training institutes. Responses were analyzed using Statistical Package for the Social Sciences, with categorical variables presented as frequencies and percentages and continuous variables as means with standard deviations.

**Results:**

A total five hudnred residents were targeted among whom one hundred residents responded, yielding a response rate of 20%. While 90% (90) rated their knowledge of brain and spinal tumors as good or excellent, only 67% (67) felt confident interpreting imaging studies. Notably, 33% (33) had received no formal training in surgical neuro-oncology. Exposure to spinal cord tumor surgeries was limited, with only 14% (14) having participated in such procedures. Mentorship and interdisciplinary collaboration were inadequate; only 20% (20) reported access to dedicated neuro-oncology mentors, and just 12% (12) rated interdepartmental collaboration as excellent. Resources were considered limited or insufficient by 69% (69) of respondents. Despite these challenges, 84% (84) supported the establishment of a dedicated neuro-oncology fellowship, and 67% (67) expressed definite interest in pursuing such training.

**Conclusion:**

This nationwide analysis highlights critical insights into the clinical exposure, training quality, and perceptions of neurosurgical residents in Pakistan regarding surgical neuro-oncology. While the majority of residents demonstrated enthusiasm and self-reported strong theoretical knowledge, substantial gaps were identified in hands-on experience, formal training, mentorship, and access to specialized resources, particularly in spinal neuro-oncology. Limited interdisciplinary collaboration and insufficient institutional support further hinder comprehensive skill development. National-level reforms are essential to align with global standards in neurooncological education and care.

## INTRODUCTION

Neurosurgery is amongst the younger fields of surgery and the development of neurosurgery is attributed to Sir William Macewen and other Americans and Europeans who took his work forward. The current advancements including, but not limited to, intraoperative imaging, neuromodulation and robotic surgery are evidence of the rate of progress of neurosurgery that has never happened before.^1^ Neurosurgery stands as the most challenging field when it comes to morbidity and mortality. Hence, a neurosurgeon should have the best of knowledge and skills that can deliver the highest quality of patient care.^2^ In order to acquire the required knowledge and skill set, the infrastructure of the training programs must be parallel to the best programs in the world. When it comes to low-middle income countries like Pakistan, where the budget allocated to the health sector is the lowest, then initiating and maintaining the quality training programs are a challenge to surmount.^2^ The GDP per capita figure for 2023 was USD 1,471.5.^3^ According to the data, there are 22 neurosurgery training centers in Pakistan with the number of residents in training mounting to 177.^4^ In a country with population boom, there is only one neurosurgeon per 720,000 people.^1^

One of the growing and evolving specialty in neurosurgery is neuro-oncology.^5^ In the recent past, surgery involving brain tumors was not considered a viable option. But, with surgical technological advancements, neuro-oncology is reshaping itself as a major sub-specialty of neurosurgery.^6^ The overall morbidity and mortality linked to central nervous system (CNS) tumors is significant. As per the Study including the Global Burden of Disease, there were 330,000 new cases and 227,000 mortalities in 2016, with an increase in incidence of 17.3% from 1990, and that too was in South Asian region.^7^ The access to neuro-oncology practice also displays certain disparities between high income countries and low-middle income countries. A survey in UK demonstrated that out of 136 neuro-oncological patients, all received surgery as the primary treatment modality. On the other hand, in Sudan, in only 8 out of 62 pediatric patients tumor resection was done and the 2-year and 5-year survival rates were 33% and 13% respectively.^8^ Pakistan also stands in the latter category.

It is an unfortunate fact that currently there is no dedicated specialty for surgical neuro-oncology in Pakistan and for the development and to integrate into neurosurgical practice, it is essential to evaluate the current neuro-oncological landscape and for this the neurosurgical residents can play a major role. If their perspectives on the neuro-oncological specialty training can be collected, then the areas to work on can be identified. Thus, with this survey, we intend to see the trends and interests of the workforce of neurosurgery in our country.

## METHODOLOGY

This nationwide, prospective observational study was conducted by Department of Neurosurgery Punjab Institute of Neurosciences, Lahore from January to March 2025, involving post-graduate residents from both public and private sector institutes of Pakistan registered with accredited residency programs. Knowledge, attitudes, and practices survey was improvised using the study documented by Madhugiri et al. in 2021.^5^ An online survey using Google Forms (Google Inc., USA). was designed in English language to collect data regarding the various domains of relevance to neuro-oncology as a subspecialty. All residents registered for the post-graduate degree programs of Fellowship in College of Physicians and Surgeons (FCPS) Neurosurgery and Masters in Surgery (MS) Neurosurgery were considered eligible to participate in the survey.

### Inclusion Criteria:

Only the residents registered with specialty training for five years under FCPS or MS programs were considered eligible to participate in the study.

### Exclusion Criteria:

Residents of other surgical specialties were excluded.

Data collectors were given the charge of collection as representatives from Punjab Institute of Neurosciences, Mayo Hospital, Sir Gangaram Hospital, Aziz Bhatti Shaheed Teaching Hospital, Nishtar Hospital, Aga Khan University Hospital, Services Hospital, Bolan Medical Complex, Sheikh Zayed Hospital Rahim, Bahawalpur Victoria Hospital, Jinnah Postgraduate Medical Centre, Dow University of Health Sciences, Hayatabad Medical Complex, Khyber Teaching Hospital and Lady Reading Hospital. Emails and messages containing the link to the survey were sent out to all residents at various neurosurgical training institutes of Pakistan including both public and private sector institutes. All demographic details, training profiles, clinical knowledge, resource allocation, surgical practices were included in data collection. At the end of the survey period, the responses were collected and analyzed. Descriptive statistics were applied and tables were generated using Microsoft Excel (Microsoft Corp., Redmond, WA).

### Data analysis technique:

Non-probability, consecutive sampling was done. This was a knowledge, attitudes, and practices survey and no new interventions were required. Statistical Package for Social Sciences (IBM SPSS Statistics for Windows. Version 24, USA) was used to analyze de-identified data. Categorical data was presented as frequencies and percentages. This included all variables with binary responses (e.g., “yes”/ “no”) or those grouped into categories (e.g., co-morbidities). Continuous/Quantitative data was presented as means with standard deviations (SDs).

### Ethical Approval:

The Institutional Review Board of Punjab Institute of Neurosciences approved the study via reference no. 2027/IRB/PINS/2025; Dated: January 23, 2025.

## RESULTS

A total 500 residents were targeted among whom 100 residents responded, yielding a response rate of 20%. The mean age of responding residents was 30.45 ± 4.46 years, with the majority of male residents (Table-I).

**Table-I:** Demographic and Training Profile of Survey Participants.

Variables	Percentage %, Frequency (n)
Mean age ± SD	30.45 ± 4.46 years
***Gender***	
— Male	72% (72)
— Female	28% (28)
***City of Hospital***	
— Lahore	69% (69)
— Multan	10% (10)
— Islamabad	1% (1)
***Sector of hospital***	
Public	94% (94)
Private	6% (6)
***Year of residency training***	
I	13% (13)
II	17% (17)
III	15% (15)
IV	22% (22)
V	33% (33)
***Prior exposure or background in oncology***	
Yes	33% (33)
No	67% (67)
Not sure	4% (4)

The study highlights that gliomas (61%) and meningiomas (45%) are the most commonly treated tumors in surgical neuro-oncology. Most respondents rated their knowledge as good or excellent, with craniotomy (84%) being the most frequent procedure. Confidence in imaging-based diagnosis was high (67%), and over half could distinguish between primary and metastatic tumors. While spinal cord tumor familiarity was moderate, gliomas and meningiomas were prioritized for surgery. Awareness of adjuvant therapies was also strong, emphasizing a multidisciplinary approach (Table-II).

**Table-II:** Clinical Knowledge and Practice Patterns in Surgical Neuro-Oncology.

Questions	Percentage %, Frequency (n)
Which of the following tumor types are primarily treated through surgical neuro-oncology?	• Gliomas 61% (61)
	• meningiomas 45% (45)
	• metastatic brain tumors 25% (25)
	• spinal cord tumors 26% (26)
	• pituitary gland tumors 25% (25)
	• acoustic neuromas 27% (27)
	• cerebellopontine angle sol 22% (22)
	• posterior fossa 21% (21)
	• intraventricular sol 18% (18)
	• olfactory sol 17% (17)
	• pineal gland sol 18% (18)
	• craniopharyngioma 23% (23)
	• 3^rd^ and 4^th^ ventricular sol 20% (20)
	• deep seated sol 17% (17)
How would you rate your knowledge of brain and spinal cord tumors and their surgical management?	• Excellent: 48% (48)
	• Good: 42% (42) • Average: 10% (10)
Which surgical procedures are commonly performed for brain and spinal cord neoplasms?	• Craniotomy: 84% (84)
	• Spinal tumor resection: 35% (35)
	• Stereotactic biopsy: 20% (20)
	• Spinal decompression: 18% (18)
	• Spinal fusion: 8% (8)
How confident are you in diagnosing brain and spinal cord tumors based on imaging studies (MRI, CT scans)?	• Very confident: 16% (16)
	• Confident: 67% (67)
	• Not confident: 17% (17)
Can you effectively distinguish between primary brain tumors and metastatic brain tumors?	• Yes: 57% (57)
	• No: 14% (14)
	• Sometimes: 29% (29)
How familiar are you with spinal cord neoplasms, including their classification (intramedullary vs. extramedullary)?	• Very familiar: 33% (56)
	• Somewhat familiar: 56% (56)
	• Not familiar at all: 11% (11)
What is your understanding of the management of primary spinal tumors, including both intramedullary and extramedullary lesions?	• Comprehensive understanding: 17% (17)
	• Moderate understanding: 49% (49)
	• Basic understanding: 29% (29)
	• No understanding: 5% (5)
In your opinion, which brain or spinal tumor types should be prioritized for surgical intervention?	• Gliomas 45% (45)
	• meningiomas 42% (42)
	• metastatic brain tumors 13% (13)
	• spinal cord tumors 20% (20)
	• pituitary gland tumors 25% (25)
	• acoustic neuromas 17% (17)
How would you rate your understanding of the importance of adjuvant therapies (radiotherapy, chemotherapy) for brain and spinal cord tumors ?	• Excellent: 17% (17)
	• Good: 52% (52)
	• Basic: 31% (31)

One-third of respondents reported no formal neuro-oncology training, though 69% frequently encounter tumor cases. Most observed brain tumor surgeries (71%), but spinal tumor exposure was limited. Only 30% had regular access to dedicated training, and resources were rated as limited by over half, highlighting key gaps in training and infrastructure (Table-III).

**Table-III:** Residency Training, Exposure, and Resource Availability in Surgical Neuro-Oncology.

Questions	Percentage %, Frequency (n)
How much formal training have you received in surgical neuro-oncology during your residency?	• Extensive (3 to 6 months): 24% (24)
	• Moderate (2 to 6 months): 22% (22)
	• Limited (a few weeks): 21% (21)
	• Not at all: 33% (33)
How frequently do you encounter brain and spinal cord tumor cases during your clinical rotations?	• Very frequently 69% (69)
	• Occasionally: 21% (21)
	• Rarely: 8% (8)
	• Never: 2% (2)
Which surgical procedures related to brain and spinal tumors have you observed or participated in?	• Glioma, meningioma, or metastatic brain tumor resection: 71% (71)
	• Spinal cord tumor resection(intramedullary, extramedullary): 14% (14)
	• Stereotactic biopsy for brain/spinaltumors: 7% (7)
	• Spinal decompression and stabilization: 8% (8)
Do you have access to dedicated training in neuro-oncology (lectures, practical sessions, online resources)?	• Yes, regularly: 30% (30)
	• Occasionally: 40% (40)
	• No, very rarely: 30% (30)
How would you assess the quality of neuro oncology training at your institution?	• Excellent: 37% (37)
	• Good: 33% (33)
	• Fair: 30% (30)
Is there a formal neuro-oncology mentorship program or specialized faculty at your institution?	• Yes, with dedicated neuro-oncology faculty: 20% (20)
	• Yes, but not focused exclusively on neuro-oncology: 45% (45)
	• No, not at all: 35% (35)
How often are the spinal cord tumor cases included in the surgical training at your institution?	• Very often: 38% (38)
	• Occasionally: 44% (44)
	• Rarely: 17% (17)
	• Never: 1% (1)
Does your institute provide access to advanced neuro-oncology tools such as intraoperative MRI, neuro-navigation, or neurophysiological monitoring for spinal tumors?	• Yes, regularly: 35% (35)
	• Occasionally: 33% (33)
	• Rarely: 32% (32)
How well does your institution integrate neurosurgery with other specialties (e.g., radiation oncology, medical oncology) for brain and spinal tumor management?	• Very well: 31% (31)
	• Somewhat well: 51% (51)
	• Poorly integrated: 18% (18)
What is the general availability of resources (staff, equipment, teaching materials) for spinal cord and brain tumor surgeries in your institution?	• Adequate: 31% (31)
	• Limited: 51% (51)
	• Insufficient: 18% (18)

Most residents rated teaching quality as good, but only 25% had specialized mentors. Research and educational resources were limited, and just 18% felt fully prepared for independent practice. A majority (64%) expressed the need for more structured neuro-oncology training (Table-IV).

**Table-IV:** Resident Engagement, Preparedness, and Training Needs in Neuro-Oncology.

Questions	Percentage %, Frequency (n)
How would you rate the quality of teaching in surgical neuro-oncology (brain and spinal tumors) at your institution?	• Excellent: 25% (25)
	• Good: 44% (44)
	• Fair: 23% (23)
	• Poor: 8% (8)
Have you been actively involved in case discussions or tumor board meetings related to brain and spinal cord tumors?	• Yes, regularly: 33% (33)
	• Occasionally: 49% (49)
	• No, never: 18% (18)
How often do senior consultants or specialized faculty provide feedback or guidance during neuro-oncology cases involving brain and spinal cord neoplasms?	• Very frequently: 37% (37)
	• Occasionally: 45% (45)
	• Rarely: 15% (15)
	• Never: 3% (3)
Is there a formal mentorship system for neuro-oncology (including spinal tumors) at your institution?	• Yes, with specialized neuro-oncology mentors: 25% (25)
	• Yes, but mentors are not specifically focused on neuro-oncology: 50% (50)
	• No, not at all: 25% (25)
How often do you attend workshops or specialized symposia focused on surgical neuro-oncology?	• Frequently: 15% (15)
	• Occasionally: 50% (50)
	• Rarely: 19% (19)
	• Never: 16% (16)
(I) Does your institution offer access to clinical or laboratory research opportunities related to brain and spinal cord tumors?	• Yes, Frequently: 23% (23)
	• Occasionally: 40% (40)
	• No, Rarely: 28% (28)
	• Never: 9% (9)
Are there laboratory research programs available for you residents for both basic and translational study of brain tumors?	• Yes: 3% (33) • No: 67% (67)
Is there dedicated research time available for you residents to complete this research?	• Yes: 39% (39) • No: 61% (61)
Are you residents involved or exposed to brain tumor clinical trials?	• Yes: 39% (39)
	• No: 61% (61)
Do you feel adequately prepared to make independent decisions in surgical management of brain and spinal cord tumors after your training?	• Yes, completely: 18% (18)
	• Yes, somewhat: 49% (49)
	• No, I need more training: 27% (27)
	• No, Not at all: 6% (6)
How would you rate the availability of neuro-oncology-specific educational materials (e.g., textbooks, online resources, articles) at your institution?	• Excellent: 19% (19)
	• Good: 37% (37)
	• Fair: 29% (29)
	• Poor: 15% (15)
Are there sufficient opportunities for you to observe or perform complex neuro-oncology surgeries involving brain and spinal cord tumors?	• Yes, frequently: 42% (42)
	• Occasionally: 38% (38)
	• Rarely: 15% (15)
	• Never: 5% (5)
Would you prefer more structured or formalized neuro-oncology training (including spinal cord tumors) during your residency?	• Yes, Definitely: 64% (64)
	• Yes, to some extent: 26% (26)
	• No, the current structure is adequate: 8% (8)
	• No, I prefer other subspecialty areas: 2% (2)

Most residents identified surgical techniques (35%) and interdisciplinary collaboration (22%) as key challenges in brain and spinal tumor management. A significant 98% reported resource shortages in Pakistan. Most respondents expressed strong interest in more spinal tumor exposure (71%) and supported the need for dedicated neuro-oncology centers (77%) and fellowship programs (84%), highlighting critical gaps and the demand for structured specialization (Table-V).

**Table-V:** Challenges, Gaps, and Future Needs in Surgical Neuro-Oncology Training in Pakistan.

Question	Percentage %, Frequency (n)
Which aspects of brain and spinal cord tumor management do you find most challenging?	• Diagnosis and imaging interpretation: 15% (15)
	• Surgical technique and approach: 35% (35)
	• Post-operative care and complications: 20% (20)
	• Multidisciplinary collaboration: 22% (22)
	• Adjuvant treatments (radiotherapy/chemotherapy): 7% (7)
	• None: 1% (1)
Do you believe there is a shortage of resources (e.g., personnel, equipment, training opportunities) for the surgical management of brain and spinal tumors in Pakistan?	• Yes, significant shortage: 55% (55)
	• Yes, some shortage: 43% (43)
	• No shortage: 7% (7)
How do you perceive the availability of research opportunities in the field of surgical neuro-oncology (both brain and spinal tumors) at your institution?	• Excellent: 18% (18)
	• Good: 31% (31)
	• Fair: 32% (32)
	• Poor: 19% (19)
Would you prefer more exposure to complex spinal tumor surgeries during your training?	• Yes, definitely: 71% (71)
	• Yes, but with some reservations: 22% (22)
	• No, I am more interested in brain tumor surgery: 6% (6)
	• No, Not interested in spinal tumor surgery: 1% (1)
Do you think there is adequate collaboration between neurosurgery and oncology departments for brain and spinal tumor management in your hospital?	• Yes, excellent collaboration: 12% (12)
	• Adequate collaboration: 36% (36)
	• Poor collaboration: 40% (40)
	• No collaboration: 12% (12)
Do you feel there is a need for more dedicated neuro-oncology centers in Pakistan, particularly for complex brain and spinal tumor cases?	• Yes, definitely: 77% (77)
	• Yes, to some extent: 14% (14)
	• No, not necessary: 9% (9)
	• No, other approaches should be prioritized: 0% (0)
Do you think that neurosurgery residents in Pakistan should have the option of pursuing a dedicated fellowship in neuro-oncology (including spinal tumors) after residency?	• Yes, strongly agree: 84% (84)
	• Yes, somewhat agree: 14% (14)
	• No, not necessary: 2% (2)

A strong majority of residents (67%) expressed definite interest in pursuing a fellowship in surgical neuro-oncology, while 66% called for more emphasis on spinal cord tumor management during training. However, 59% reported limited to no international exposure or collaboration, highlighting a key area for improvement in global engagement and learning opportunities (Table-VI).

**Table-VI:** Fellowship Aspirations and International Exposure in Neuro-Oncology Training.

Question	Percentage %, Frequency (n)
Would you be interested in pursuing a fellowship in surgical neuro-oncology (including spinal tumors) after your residency?	• Yes, definitely: 67% (67)
	• Maybe, depending on the opportunity: 30% (30)
	• No, I am interested in other subspecialties: 3% (3)
Do you think there is sufficient international exposure or collaboration in the field of surgical neuro-oncology (brain and spinal tumors) for residents in Pakistan?	• Yes, plenty of opportunities: 12% (12) • Some opportunities: 29% (29)
	• Very few opportunities: 48% (48)
	• None at all: 11% (11)
Do you think there should be more emphasis on the management of spinal cord tumors during neurosurgery residency training?	• Yes, definitely: 66% (66)
	• Yes, somewhat: 27% (27)
	• No, it is adequately covered: 4% (4)
	• No, other areas should be prioritized: 3% (3)

## DISCUSSION

This study was conducted to evaluate the clinical exposure, perception, and training experience in surgical neuro-oncology among neurosurgery resident trainees across Pakistan. Understanding the preparedness of trainees in such a critical and evolving field is essential for advancing patient outcomes and strengthening neurosurgical training frameworks. As global neurosurgical education shifts towards more specialized and multidisciplinary approaches, especially for brain and spinal tumor management, it becomes increasingly important to assess where Pakistan stands.^9^ The growing burden of neuro-oncological diseases and the lack of comprehensive specialty care in LMICs, this study fills a major gap by providing a national perspective.^10^,^11^

The majority of survey respondents were residents from public-sector hospitals 94% (94), with most responses coming from Punjab 80% (80), primarily from Lahore 69% (69). This distribution closely mirrors historical patterns noted by Javed et al., who documented that most neurosurgery training hubs, such as Punjab Institute of Neurosciences and Jinnah Postgraduate Medical Centre, are concentrated in major urban centers.^12^ Shamim et al., similarly observed that public urban hospitals dominate neurosurgical education, with major provinces like Balochistan and Gilgit-Baltistan remaining critically underserved.^13^

This urban-centric concentration reflects systemic healthcare inequities that persist in Pakistan and highlights the importance of decentralizing specialty training. While the residents demonstrated a high level of self-reported confidence regarding their theoretical knowledge of brain and spinal tumors, our findings revealed significant gaps when assessed on clinical and imaging skills. Although 90% (90) rated their knowledge as good or excellent, only 67% (67) felt confident interpreting imaging studies. A similar trend was observed in previous national surveys such as those conducted by Ali et al., and Shamim et al., where residents showed a disparity between theoretical comprehension and practical diagnostic capability.^13^, ^14^ This mismatch can be attributed to the limited number of structured neuroradiology meetings, case discussions, and supervised imaging sessions in many Pakistani institutions.

Our study further found that approximately one-third 33% (33) of the participants reported receiving no formal training in surgical neuro-oncology during their residency. This mirrors prior findings by Javed et al., who emphasized the lack of a standardized, updated curriculum focused on neuro-oncology in Pakistan.^12^ Similarly, Shamim et al., noted that 40% of centers lacked regular academic activities such as journal clubs and neuropathology meetings, critical for reinforcing specialized knowledge.^13^ However, compared to data from earlier periods, a minor improvement was seen in our survey, as 30% (30) residents reported regular access to dedicated training resources, potentially reflecting recent efforts to enhance academic activities at selected high-volume centers.^12^

The study also revealed poor mentorship structures and weak interdisciplinary collaboration, with only 20% (20) of residents reporting access to dedicated neuro-oncology mentors. Ali et al., and Kiran et al., both stressed the profound absence of structured mentorship and interdepartmental cooperation in Pakistan’s neurosurgery training system.^14^,^15^ Residents often lacked consistent guidance from subspecialty-trained faculty, which in turn limited their exposure to comprehensive brain and spinal tumor management strategies.^14^ Furthermore, only 12% (12) of respondents rated neurosurgery-oncology collaboration at their hospital as excellent, highlighting the scarcity of integrated tumor boards and collaborative care pathways discussed in a study done by Barbaro et al.^9^

Regarding resources and research opportunities, a large proportion of trainees reported inadequate access. About 51% (51) reported limited resources, while 18% (18) described them as insufficient. This significant gap aligns with previous observations made by Shakir et al., who described the chronic underinvestment in neurosurgical infrastructure across LMICs.^10^,^11^ Shamim et al., also highlighted that 95% of training centers lacked microsurgical laboratories, and fewer than half subscribed to multiple indexed neurosurgical journals, severely restricting academic growth.^13^

In the context of spinal tumors, our study highlights that spinal neuro-oncology remains a neglected area within neurosurgical residency training. Only 14% (14) of residents reported hands-on exposure to spinal cord tumor surgeries. This is consistent with previous findings by Javed et al., who pointed out that complex spinal tumor surgeries receive little emphasis compared to cranial procedures.^12^ Additionally, 66% (66) of residents strongly felt that spinal tumor management deserved greater attention in the training curriculum. This imbalance deprives residents of crucial surgical competencies necessary for managing spinal neoplasms, further widening the gap between Pakistan’s neurosurgical education and international best practices.

Encouragingly, our data showed that a majority of residents expressed strong interest in pursuing a dedicated fellowship in surgical neuro-oncology after residency, with 84% (84) supporting this initiative. Furthermore, 67% (67) expressed a definite personal interest in undertaking such a fellowship if available. Such enthusiasm was also reported in global neurosurgical education literature like Sundar et al., which emphasized the evolving expectations of neurosurgical trainees who seek structured, subspecialty-focused training in areas like oncology, machine learning, and quality improvement.^16^ In contrast to earlier findings by Shamim et al., where fellowship interest was lukewarm, our results reflect a new generation of residents more attuned to international educational trends and academic career planning.^13^

This nationwide analysis highlights a complex picture, while residents show commendable enthusiasm and self-assurance, significant systemic gaps persist in surgical neuro-oncology training in Pakistan. Comparisons with previous studies reveal persistent deficiencies in resources, mentorship, interdepartmental collaboration, and structured academic exposure. Although some modest improvements have occurred, the overall training environment still requires substantial reforms. Investment in dedicated neuro-oncology programs, mentorship structures, research opportunities, and better hospital collaboration is critical if Pakistan hopes to match global standards in surgical neuro-oncology in the future.

### Limitations:

This study has several important limitations that should be acknowledged. Firstly, the sampling method used was non-probability and case series based, which affects how well the results can be applied to all neurosurgery residents across Pakistan. A large number of responses were from Lahore, especially from the Punjab Institute of Neurosciences, so the data may not reflect the experiences of residents from other regions.

There was also a strong bias toward public sector institutions, with 94% of participants coming from government hospitals, which may leave out key insights from private setups. Since the information was entirely self-reported, it’s possible that some responses were influenced by personal bias, either intentionally or unintentionally. The study also lacked objective assessment of surgical skills or knowledge relying only on perceived confidence and self-evaluation. Many residents reported limited exposure to certain procedures, especially spinal tumor surgeries, which could affect their views on the subspecialty. Access to advanced tools, mentorship, and research opportunities also varied, which may have shaped perceptions.

The survey was only open from January 15 to March 31, 2025, so some residents may have missed it. It also focused solely on residents, excluding faculty, administrators, and patients who could have added valuable context. Lastly, its cross-sectional design limits insight into how perceptions might change over time or with training improvements.

Future research should use probability-based sampling to better represent residents across regions and private institutions, include objective assessments of surgical skills, and capture perspectives of faculty, administrators, and patients. Longitudinal studies are also needed to track how training initiatives and access to advanced tools impact resident perceptions over time.

### Recommendations:

**Fig.1:**
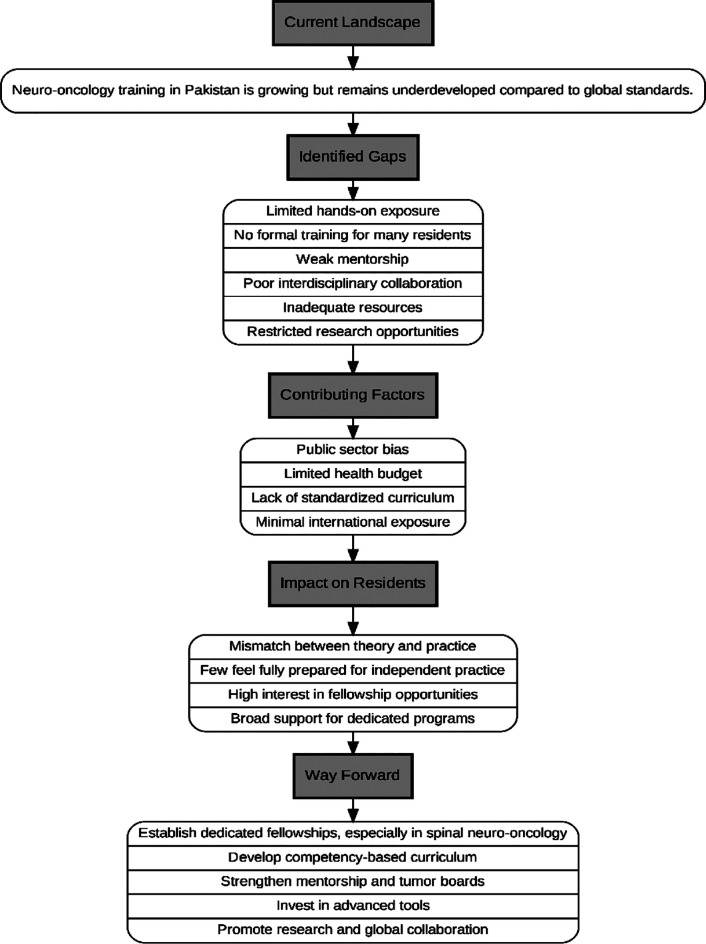
Author-proposed illustration of Clinical Landscape and Perception of Surgical Neuro Oncology among Neurosurgical Resident.

Authors propose the way forward in Fig.1. To address gaps in surgical neuro-oncology training, we recommend establishing dedicated post-residency fellowships, particularly in spinal tumor surgery, reflecting the interest of 84% of residents. Spinal neuro-oncology should be incorporated into core training through mandatory rotations and surgical exposure. A standardized, competency-based curriculum should be implemented nationwide under national residency programs to ensure uniformity. Strengthening mentorship by appointing dedicated neuro-oncology mentors is vital, as over 75% of centers currently lack such support. Interdisciplinary integration should be promoted via regular tumor boards and multidisciplinary meetings. Investment in advanced tools like intraoperative imaging, neuro-navigation, and neuromonitoring is needed, especially in under-resourced settings. Research should be encouraged through protected time, lab access, and support for clinical trials. National and international collaborations via exchanges and observer-ships will help align local practices with global standards. Expanding training opportunities beyond urban areas will reduce geographic disparities, while centralized repositories of lectures and surgical videos will provide equitable access to high-quality resources.

## CONCLUSION

This nationwide analysis highlights key insights into the clinical exposure, training quality, and perceptions of neurosurgical residents in Pakistan regarding surgical neuro-oncology. While most residents showed enthusiasm and reported strong theoretical knowledge, major gaps were noted in hands-on experience, formal training, mentorship, and access to resources, especially in spinal neuro-oncology. Limited interdisciplinary collaboration and weak institutional support further hinder skill development. Encouragingly, many trainees expressed strong interest in fellowships, underscoring the need for structured subspecialty programs, better infrastructure, and national efforts to align with global standards.

### Authors’ Contribution:

**SHH, TS, IKI, ZT, AM:** Data acquisition and data interpretation, drafted manuscript.

**HMQ:** Concept and design of the stud, critical review of manuscript and supervision of the study,

**RS:** Data acquisition and data analysis, drafted the manuscript.

All authors agree to the final version of the manuscript to be published and be accountable for all aspects of the work.
